# Living with Discordance: A Qualitative Description of the Challenges Faced by HIV Negative Married Women

**DOI:** 10.30476/IJCBNM.2020.82845.1093

**Published:** 2020-04

**Authors:** Mona Larki, Narjes Bahri, Javad Moghri, Robab Latifnejad Roudsari

**Affiliations:** 1 Student Research Committee, Department of Midwifery, School of Nursing and Midwifery, Mashhad University of Medical Sciences, Mashhad, Iran; 2 Department of Midwifery, School of Medicine, Social Development & Health Promotion Research Center, Gonabad University of Medical Sciences, Gonabad, Iran; 3 Social Determinants of Health Research Center, Mashhad University of Medical Sciences, Mashhad, Iran; 4 Nursing and Midwifery Care Research Center, Mashhad University of Medical Sciences, Mashhad, Iran; 5 Department of Midwifery, School of Nursing and Midwifery, Mashhad University of Medical Sciences, Mashhad, Iran

**Keywords:** Challenge, HIV/AIDS, Iran, Qualitative description, Serodiscordant couple

## Abstract

**Background:**

Serodiscordant couples are faced with many social, sexual and relationship challenges in all aspects of their lives. The sources of conflicts could be disease acquisition, transfer of HIV to the uninfected partner, and fertility decisions. The current qualitative study was designed to explore the challenges faced by HIV negative women in serodiscordant relationships.

**Methods:**

This qualitative description was conducted in Mashhad, Northeast of Iran, between October 2018 and June 2019. 15 HIV-negative women who were living with their HIV-positive husbands were selected through purposive sampling method. The data were collected using semi-structured interviews. Data were analyzed using conventional content analysis adopted by Graneheim and Lundman. MAXQDA version 12, was used for data organization. Components of rigor including credibility, dependability, confirmability and transferability were considered.

**Results:**

The main overarching theme which emerged from the qualitative study was “threats to family life”, consisting of five categories along with their subcategories. These categories included stigmatic reactions followed by disclosure of the status, social misconceptions and limitation of information sources, psychological disruptions, hard decision making for fertility, and role conflict in the family.

**Conclusion:**

This study provides an insight into different aspects of challenges faced by Iranian women in HIV-serodiscordant relationships. Also, our study supports the view of other investigators who believe that there is an urgent need for provision of counseling and empowerment interventions for HIV- serodiscordant couples.

## INTRODUCTION

HIV/AIDS is the second most common cause of mortality among communicable diseases, and it is estimated that 37.3 million people are living with HIV in the world. ^1^ Iran with an estimated 66000 people living with HIV ^2^ is one of the high-risk countries for AIDS in the Middle East region. ^3^ Following the development of the use of antiretroviral treatments in seropositive patients, life expectancy has increased. ^4^ Also, because of the viral load suppression, ^5^ the desire for marriage and childbearing has dramatically increased. ^6^ Thus, the researchers have suggested that the number of serodiscordant couples are increasing. ^7^ HIV serodiscordant couple refers to the mixed-status and situation where one of the partners is HIV positive, while the other is HIV negative. It is estimated that 75% and 37% of serodiscordant couples are living in the countries with low and high HIV prevalence countries, respectively. ^8^ Globally, half of all people with HIV are in long-term and regular relationships with the HIV-negative partners. ^9^ They are a priority population in prevention of HIV transmission through sexual contact. ^10^ The conceptualization of the issue of HIV/AIDS serodiscordant couples is difficult; also, there is a poor understanding of it in couples and among most health care providers. ^11^

These couples are faced with many social, sexual and relationship challenges in all aspects of their lives. The sources of conflicts, anxiety and concerns could be disease acquisition, transfer of HIV to the uninfected partner and fertility decisions. In serodiscordant couples, the HIV negative partner is at high risk of HIV infection; age adjusted rate ratios can be as high as 12 for males and 106 for females compared with couples in relationships where both partners are HIV-negative. ^12^ Therefore, a key challenge for them is decreasing the risk of HIV transmission to their negative partner(s) and to any children conceived. ^13^ The results of a study in Ghana showed that the main challenges for serodiscordant couples include fear of infecting their children, economic pressures, irregular use of condom, and death. ^14^

The results of a qualitative study in serodiscordant couples in Uganda showed that in addition to focusing on biomedical and behavioral aspects, interventions must also address the cultural complexity, understanding the context and dynamics of the relationships between couples. ^15^ Most studies have been conducted on serodiscordant couples in African countries. The focus of these studies is mainly on issues such as HIV risk perceptions, male partner support, trust between partners, communication issues, decision-making processes within relationships, community norms surrounding biomedicine, and HIV-related stigma. ^16 , 17^ Given that HIV serodiscordance phenomenon is not well understood and there are many misconceptions about it, ^18^ quantitative methods cannot explain the damage caused by long-term confrontations in families with a member of HIV positive. However, qualitative approaches are robust methods of inquiry which could clarify the ambiguous and unknown dimensions of the phenomenon under the study. ^19 , 20^ Also, qualitative inquiries are useful and rigorous methods for investigating sensitive topics such as HIV. Besides, they provide better understanding of the manifest and latent meanings embedded in the context. ^21^

Despite the vast number of available surveys about people living with HIV, research on the challenges of serodiscordant individuals is sparse and has largely focused on patients. Hence, heterosexual serodiscordant couples represent a key subpopulation for HIV prevention but are not well studied in Iran. Therefore, this study aimed to investigate the challenges faced by HIV uninfected women in the context of HIV serodiscordant relationships. 

## MATERIALS AND METHODS

We used a qualitative descriptive study. ^22^ For the purpose of this study, we chose a Clinic of Behavioral Disorders Counseling in Mashhad, Iran, for study. The reason for the selection of this clinic was that it was located in the central part of Mashhad, Iran and all patients with HIV and their wives were accessible and their health documents were available for the study. The study was conducted from October 2018 to June 2019. The researcher contacted the women with HIV positive husband to obtain their experiences of the challenges they faced with in their lives. The inclusion criteria for the study were women with HIV positive husbands, the ability to talk and express emotions and feelings, and the willingness to participate in the study. The exclusion criteria was unwillingness of the participants to continue the study. 

The purposive sampling method was used to select the sample for the study. All interviews were conducted by the first researcher, who is a midwife researcher with previous experience of interviewing with women with risky sexual behaviors in prison. The interview continued with an open-ended and general question: Could you please explain your experiences of living with an infected husband to HIV? The interview continued with some more specific questions like: Could you explain one of your problems in living with your husband? Probing questions were also used during the interview, such as: Can you give me an example? Also, during the interview process, researcher considered non-verbal cues such as body language and facial expressions. All the interviews were tape recorded and transcribed. Interviews lasted between 50-70 minutes. The participants consisted of 15 married women. One interview was conducted with each participant. The profile of participants is shown in Table 1. Sampling was continued until data saturation. We reached data saturation when no other new concept generated from the last three participants.

**Table1:** The participants’ profile (N=15)

Participant	Age(Years)	Duration of marriage (years)	Education	Employment	Husband’s occupation	Children (n)	Economic status	HIV -disclosure to relatives	Elapsed time since disclosure (years)	Consistent condom use	Perceived childbearing desire
1	36	18	7 years of schooling	Housewife	Temporary employees	2	Poor	Yes	4	No	No
2	38	5	High school diploma	Laborer	Employed	-	FA^a^	No	5	Yes	No
3	34	8	9 years of schooling	Seller	Temporary employees	1	Poor	Yes	7	No	Yes
4	45	30	5 years of schooling	Tailor	Unemployed	3	FA^a^	No	6 months	No	No
5	30	15	5 years of schooling	Housewife	Owned private businesses	1	FA^a^	No	12	Yes	No
6	34	12	9 years of schooling	Housewife	Temporary employees	1	Poor	Yes	4	No	No
7	37	13	4 years of schooling	Housewife	Temporary employees	2	Poor	No	10	Yes	No
8	39	10	8 years of schooling	Housewife	Temporary employees	1	Poor	Yes	8	No	No
9	35	14	6 years of schooling	Housewife	Temporary employees	2	Poor	Yes	6	Yes	No
10	41	20	7 years of schooling	Housewife	Temporary employees	3	Poor	No	10	No	No
11	40	19	8 years of schooling	Housewife	Temporary employees	4	Poor	No	5	No	No
12	36	14	6 years of schooling	Laborer	Owned private businesses	2	FA^a^	No	6	No	No
13	35	7	High school diploma	Housewife	Unemployed	4	Poor	No	3	No	No
14	42	21	8 years of schooling	Housewife	Unemployed	2	Poor	No	6	Yes	No
15	60	35	High school diploma	Housewife	Owned private businesses	2	Good	No	4	Yes	No

a Fairly appropriate

 All interview sessions were conducted based on the willingness of participants in a private room at the clinic of behavioral disorders counseling. All interviews were audio-recorded, transcribed and entered into MAXQD version 12 that was developed and distributed by VERBI Software based in Berlin, Germany. Data analysis began immediately after the first interview. We used the Graneheim and Lundman’s (2004) method to analyze the data. ^23^ At first, the text of the interviews was read several times to obtain a general understanding and sense of the whole of their content. Then, the text of each interview was divided into meaningful units as words, phrases, sentences, and paragraphs. The meaning units were condensed and labeled with code. Then, the content codes were compared in terms of similarities and differences, and the similar codes were placed in the initial categories and then transformed to subcategories. Finally, with the advancement of data analysis, subcategories were developed to categories and themes.

In this study, credibility was established through interview with informant participants and appropriate sample size. The observational notes contained the researcher’s thoughts and feelings, impressions, as well as the interpretation of the interviewees’ non-verbal cues and body language. Also, the supervisors reviewed the processes of interviewing, coding, categorizing, and interpreting the findings. For dependability, the researcher gave data to an independent researcher who was skilled in the field of qualitative research to do an independent examination of the data and confirm them. To ensure confirmability, three reviewers reviewed the decision trail of the study as well as findings, interpretations, and conclusions of the study. For transferability, there was an attempt to present a clear description of the culture, context, sampling, and characteristics of the participants, and the process of data collection and analysis. 

The researcher gave an opportunity to participants to ask any questions about the subject under study during the interview. Before the end of interview, each participant was asked for agreement to be contacted again in the future, if necessary. All the participants agreed about it and gave their phone number to the researcher. All participants volunteered to take part in the study subject to explanations they received about the objectives and benefits of the study, as well as confidentiality and anonymity of information they were going to give. All participants were given informed consent to read and sign. In addition to verbal consent, written consent was obtained from the participants. All information collected from women was kept confidential and anonymous, i.e. instead of using their names, women were given codes to be used in the analysis. Also, it was emphasized that they can withdraw any time from the study. If the participant was not comfortable to answer any question, she was not forced to answer that. At the end of the interview, a gift was given to the participants for appreciation. Ethical clearance for this study was obtained from ethics committee of Mashhad University of Medical Sciences (Code of Ethics. IR.MUMS.NURSE.REC.1397.022).

## RESULTS

The respondents’ age ranged from 30 to 60 years. Five female’s husbands 5 (33.33%) interviewed in this study were HIV positive and HCV positive, whilst 10 (66.66%) were only HIV positive. Except for one couple, all of them had children. The majority of the females were in childbearing age when they discovered their status. Most of the females completed lower secondary school education and three females had a diploma. The majority of the females chose not to disclose their status to their children. Only one woman informed her children and one informed her entire family. 

Results of the study included 106 meaning units, 193 codes, seventeen subcategories, five categories and one theme as *“threats to family life”*, which consisted of five categories along with their subcategories. These categories included stigmatic reactions followed by disclosure of the status, social misconceptions and limitations of information sources, psychological disruptions, hard decision making for fertility, and role conflict in the family (Table 2), which are discussed below.

**Table2:** Theme, categories and subcategories extracted from the study

Subcategory	Category	Theme
Dissolution in interpersonal communication	Stigmatic reactions followed by disclosure of the status	Threats to family life
Prejudice against patients and their family		
Discrimination in relation to equal treatment		
Misconceptions about serodiscordant couples in community	Social Misconceptions and limitation of information sources	
Limited access to explicit information		
Lack of negotiation skills for safe sex practices		
Anger	Psychological disruption	
Loss of control		
Grief		
Fear		
Shock and confusion		
Fear of being infected and dependent on others	Hard decision making for fertility	
Concern about HIV transmission from mother to child		
Dealing with uncertain future		
Priority of husbands’ health needs		
Neglecting the role of husband	Role conflict in the family	
Failure of paternal function		

### *1. Stigmatic Reactions Followed by Disclosure of the Status*

Women due to distorted privacy followed by disclosure of the status preferred to keep this issue as a secret in their life. In fact, the challenge arising from the men’s status affected their wives.

1.a. Dissolution in interpersonal communication: Being neglected by families was stated by many participants. Also, none of the participants experienced support from their friends and family following the disclosure. One of the participants stated this challenge: *“What are the benefits of informing relatives? I experienced the rejection of my husband from the family. I do not like my husband to be treated with disrespect among my relatives, so I will not expose his illness to other relatives.” (P15)*

1.b. Prejudice against patients and their family: Judging by people was an important barrier to receiving services for women. The following statements confirm this: *“People think that men with AIDS are unfaithful to their wives. Those who are aware of my husband’s status think that I and my child are infected with AIDS also.” (P7)*

1.c. Discrimination in relation to equal treatment: One of the main issues raised by most of participants was lack of humanized relationship and not being discriminated by the health authorities in the health sectors. 

A woman who had a good relationship with her husband said: *“For example, when he was ill the hospital where we referred for care, they said loudly that he is HIV positive. Also, a nurse who took care of my husband violently when I protested to him; he said with a bad tone, “your husband is HIV positive I should take care of myself”.” (P2)*

### *2. Social Misconceptions and Limitation of Information Sources*

Nearly, all participants noted the lack of accurate understanding of serodiscordant status in the community. 

2.a. Misconception about serodiscordant couples in community: Some participants highlighted that providing information should not be in a way that it creates fear and unclear understanding about serodiscordant status in the community. One participant stated: *“When I was informed of my husband’s illness, I thought that, of course, I’ve been infected with AIDS, and might die soon…” (P8)*

2.b. Limited access to explicit information: The women expressed the responsibility of the government, media and health care systems to provide explicit information about HIV/AIDS to the community members. One participant explained: *“I had only seen AIDS posters in healthcare service centers. AIDS was equal to death for me. In my opinion, putting appropriate books in the park or bus is necessary to raise public awareness.” (P13)*

2.c. The lack of negotiation skills for safe sex practices: Women emphasized that condom use is an effective method to protect them, but sometimes their husbands do not want to use it. It was a struggle for them. One participant described: *“He was reluctant to use a condom. Often, he threatens me when he does not use it. I can’t negotiate with him because I have no skill of negotiation; it is difficult to convince him.” (P1)*

### *3. Psychological Disruption*

Initially, participants experienced negative emotions and mixed feelings. Some women who were interviewed said that most of the destructive psychological reactions were due to misconceptions about HIV.

3.a. Anger: Most of the participants became aware of their husband’s HIV-positive status when they were very sick and showed severe symptoms of HIV. None of the women knew their husband status before marriage. This secrecy led to anger after being informed. One of participants in this regard told: *“When my husband was hospitalized, he still hided his status. My mother- in- law knew he was infected with AIDS, but told me he had cancer. The telling lie to me made me so angry.” (P12)* However, some participants became aware of their husband status, accidentally. One woman described her awareness as follows: *“We were married for about 2-3 months. Several times, I saw my husband using drug. I asked him what are you taking? Then, he described his situation for me … I got very angry”. (P2)*

3.b. Loss of control: Informing about differences in HIV status has led to the loss of control in decision-making about the divorce or continuing living with their husband by women. This quote reflects it: *“in this case, I did not know what to do? I was pregnant. I did not know whether to separate or to live with him. In addition, I wanted to commit suicide because I was very afraid of myself and my kid to be victims of AIDS.” (P7)*

3.c. Grief: Another reaction that participants stated when faced with their husband’s status was grief. Grief due to serodiscordant status was frequently stated. Below is quotes from a participant: *“My husband knew about his status before we got married. He thought if I am informed of his status, I would not marry him. However, after discovering that we were serodiscordant, I felt comfortable, but I was sad about loss of our previous relationship. I was constantly crying…” (P8)*

3.d. Fear: Fear and concern were the most common feelings experienced by the participants following awareness about the situation. The source of fear in them was due to stigmatization and death of husband. One woman explained the reaction as follows: *“I was pregnant when I was informed of my husband’s status. The fear of this situation and rejection by people, as well as dying in a very bad condition, would increase my fears.” (P7)*

3.e. Shock and confusion: Women could not understand how it might be possible that they stay uninfected after having sexual relationships without using any protective device. One participant stated that: *“When I noticed my husband’s status, I was shocked… because I never thought that my husband was infected with AIDS because I had absolute trust in him” (P2)* (Lip biting). (She began biting her lips while expressing her experience. Repeated biting of the lips indicated stress and emotional tension associated with recalling past experiences). 

### *4. Hard Decision Making for Fertility*

The results of the interviews showed that in HIV-serodiscordant relationships, fertility desire mostly is neglected due to the risk of transmission of HIV to the negative partner and child and socioeconomic problems such as loss of economic support. 

4.a. Fear of being infected and being dependent on another one: the desire for childbearing among HIV serodiscordant couples was low because they faced with a concern about being infected themselves. Women were fearful of being infected. One woman explicitly stated: *“Personally, I believe it is better not to have any children in this condition. My husband believes that not getting infected with AIDS (protection from infecting me) is more important than wanting a kid. He says if you become infected, who wants to take care of me? Fear of infecting is always with me.” (P2)*

4.b. Concern about HIV transmission from the mother to child: The majority of participants thought their children were in risk of acquiring the HIV infection even if they used safe conception methods. One participant explained: *“The doctor told the probability of having a healthy kid by the existing methods is very high for you. But I’m so scared, even if there is one percent chance of HIV transmission to my child, I never prefer to get pregnant.” (P2)*

4.c. Dealing with uncertain future: Some participants had the opinion that failure to meet the needs of individuals in the community from one hand and the husband’s illness, on the other hand, has led to women’s unwillingness to have a child or make decision to have more children. One of the women mentioned: *“Unfortunately, at present, the economic and social problems in the community have grown a lot. My husband does not have a stable job and is not a good, decent source of income, so I think if we want to be a kid again, the child cannot have any good future.” (P14)*

4.d. Priority of the husband’s health needs: For some women, meeting the needs of the husband and taking care of him was more important than childbearing. One of the interviewees explained that: *“I truly believe that it is my duty to look after him… My husband’s illness is such that I must pay much attention to his physical and emotional needs. I’m worried that if I have a kid, I cannot respond properly to his needs.” (P2) *

### *5. Role Conflict in the Family*

Being rejected from the workplace and having imbalance in the husband’s physical health could cause a negative effect on the women’s mental and physical health. This challenge is also directly attributable to the status of the men, which led to the challenges experienced by women. 

5.a. Neglecting the role of husband: The lack of attention to the physical and psychological needs of women by the husband was another problem for participants. One respondent expressed: *“My husband is angry with me all the time. He is unemployed and does not ask about our living expenses. For example, if I want to buy something he never agrees. He doesn’t pay for the home expenses... I really need his attention.” (P4)*

5.b. Failure of paternal function: Lack of intimate relationship between husband and children and meeting their needs alone were expressed by some women. One of the women indicated that: *“My husband is reluctant to spend time with our kids. I take care of kids and try to give them relief as well, but is it my responsibility only? He doesn’t know about father`s duty. This issue makes me very sad.” (P5) *

## DISCUSSION

This qualitative study sought to understand the challenges faced by HIV uninfected women in their lives in the context of HIV serodiscordant relationships. The key findings that emerged from the data analysis indicated that HIV-uninfected women after awareness of the issue of their sero-status difference face with complex problems in their life course. In this study, various challenges unique to women were identified after diagnosis of the serodiscordance status. They are broadly categorized as encountering with stigmatic reactions followed by disclosure of the status, social misconceptions and limitations of information sources, psychological disruptions, difficult decision-making for fertility, and role conflict in the family. Given that these numerous challenges are interlinked with personal, interpersonal, socio-cultural, and structural issues; therefore, women with different conditions experience these challenges differently. However, in general, challenges threaten the health of women and also affect all aspects of their social life. In addition, the findings imply that serodiscordant relationship is not recognized in the community as a ‘norm’; therefore, all people need to be aware of serodicordant status in order to understand and accept this type of relationship.

Some women who were interviewed claimed that the disclosure of the husband’s status was associated with the negative consequences. These negative consequences caused the couples to be reluctant to disclose their status. Although, several studies have been conducted on the experiences of stigma in individuals with HIV, ^24 - 26^ the researchers have not found, so far, any study in Iran that report stigma in Iranian women in serodiscordant relationships. In our study, they experienced different forms of stigma after informing the community. The disclosure of status is an issue that affects only HIV positive individual, but in serodiscordant relationships, it can affect both partners. The consequences of disclosure in several surveys included family members’ breakage, loss of friends, and child stigma. ^27 , 28^ Generally, disclosure is an opportunity for empowering actions that assist the patients in receiving support, care and relief for emotional suffering. ^25^ However, in our study the lack of a supportive system led to the unwillingness of women to disclose the status of their spouse to other people. In contrast to our results, it was reported that one negative partner disclosed the difference in their serostatus to the family, and the family accepted the partner’s status, so that they found it easy to disclose it to the friends and others. ^29^ Given that our study was conducted in Iran, religious contexts in the community could serve as a barrier to HIV interventions and educational programs. This impediment leads to a lack of awareness about the way the virus is transmitted, the approach to prevention of transmission, and the extent of irrational fears leading to stigma and discriminatory attitudes towards people living with HIV.

Discrimination in the health system was another serious problem for our participants. Therefore, they had the experience of insult, humiliation, negligence, and lack of accountability when going to health centers for their husband’s problems. It was also an enormous barrier on women’s referral to the clinic and getting the HIV counselling and testing services. 

Other studies in Iran have also shown that physicians and nurses involved in the issues of HIV patients had similar reactions. ^24^ Because of the Islamic rules in Iranian society and the close ties between HIV and illicit sexual relationships, the women’s husbands may be charged due to unlawful sex. This painful charge could be an underlying cause for the development of stigma against them. If discrimination against patients in the health sector continues, patients and their families will have to hide it, which will increase the spread of AIDS. For this reason, healthcare policy makers are advised to pay attention to this major issue. 

The social misconceptions and limitation of information sources was experienced by many participants in this study. They referred to the important role of the media and the government in providing complete information to the public. This finding is consistent with a ten-year research in Asia, Africa, and Latin America. In these studies, it was concluded that the media play an important role in the prevention of AIDS by providing accurate information. ^30^ A possible explanation for the similarity of the results could be that both studies were done in resource-limited settings where individuals do not have accurate information about HIV. 

Based on the results of our study, negotiation for safe sex was a major problem in the lives of serodiscordant couples. It was evident in our study that participants’ husbands insist on having sex using a condom. This might be due to such issues as low risk perception and lack of awareness about HIV. A qualitative study conducted in Kenya on serodiscordant couples reflects our finding that this group faced challenges to consistent and correct condom use. ^31^ Based on a study, the issue of using a condom in serodiscordant couples is not the highest at the start of the relationship or in sporadic relationships. This finding leads to concerns about the unimportance of safe sex behaviors in this population. ^32^ As shown in our study, some of the scenarios expressed by women indicated their inability to convince their husbands to use condoms. Lack of power in women to negotiate in this case can be due to religious or spiritual beliefs and cultural norms regarding women’s obedience of men in civil society. The finding may also be due to low levels of formal education and socio-economic status of women. 

Another major challenge in serodiscordant relationship was psychological reactions in dealing with the issues of difference in sero-status. Psychological reactions affect the process of women’s coping with HIV/AIDS of the husband. Based on a study, one positive and one negative member in their relationships experienced psychosocial stresses such as anger, guilt, hopelessness, symptoms of depression, anxiety and, in some cases, suicidal ideation. ^11^ The type of psychological reactions to the husband’s illness can be associated with previous interpersonal relationships with them. In the serodiscordant couples, there is no continuous participation of uninfected partner in the care process, and the main focus is on preventing HIV transmission. The data obtained indicate that health care workers should provide appropriate psycho-spiritual counseling and support when dealing with such women.

One of the most important challenges that many participants expressed was hard decision making for fertility. However, for these couples (HIV-infected male and HIV-uninfected female), who desire for childbearing, there are optimal and harm-reduction strategies to get pregnancy associated with the transmission risk, treatment efficacy, and affordability. ^33^ Although they are often advised not to become pregnant, many factors such as economic, cultural and social issues motivate many couples to reproduce. ^34^ We found out that women, who narrated their experiences, wished to continue their marital relationships despite their husband’s HIV status, but without any decision for having a child or more children. In contrast to our results, a study showed the women’s tendency to maintain their relationship as well as have children in the near future. ^35^ The reasons for this difference could be the differences in contextual factors such as the sociocultural issues of the areas under the study and the characteristics of the participants. For instance, in a study carried out in Kenya, it was reported that due to the high prevalence of HIV, serodiscordant couples were considered relatively normal. In Kenya, informing about safe conception methods is more than Iran. Furthermore, in addition to women, men in serodiscordant relationships were included in the study. ^10^

In a study carried out on HIV-serodiscordant couples in Kenya, it was found that some health care providers had limited knowledge about safer conception methods and frequently prohibited HIV serodiscordant couples from natural conception. ^36^ This finding was not consistent with those of our study. In our study, the participants explained that health care providers did not have a poor understanding of the conception of these couples and thus encouraged them to become pregnant. 

Men’s lack of attention to parental and marital duties was another challenge posed by women. The anticipation of chronic illnesses results in loss of individual performance in the family. Our study showed that HIV affects the economic status and employment opportunities. Men, due to financial resource and income impoverishment, did not have the ability to meet family needs. According to the results of a study, the loss of employment occurs for HIV-affected persons within 1 year after HIV infection. ^37^ Our results were in line with a negative link between HIV and unemployment. AIDS makes a financial burden on the patients and their families. The reasons for the financial burden can be the costs of treatment or the loss of productivity. ^38^ Financial burden in HIV-affected families may lead to creating new forms of relationships and roles. 

Notwithstanding the limitations of this study, there are several strengths to this study. The studies conducted about HIV/AIDS have mostly focused on the HIV positive individual rather than the HIV negative ones, but we conducted the study using the qualitative method to obtain the viewpoints of HIV-negative women among HIV serodiscordant couples. Also, numerous investigations have explored the needs and challenges of people with HIV, in Iran, but the challenges that affected the persons’ experience in their lives have not been adequately addressed. ^12^

There are several limitations to the interpretation of our findings. Recruitment for this study was limited to a large counseling center behavioral disorders in Mashhad using a purposive sampling on a population of couples referring to it, and other couples who did not refer to the clinic were not interviewed. Therefore, the findings are only the experiences and reflections of serodiscordant couples within this context. Another limitation of the present study was the lack of precise knowledge of how men were affected by HIV. The way that husbands have been infected can be an important source of challenges and conflicts experienced by women. However, in this study, women did not know exactly how their husbands were infected. Since this was a qualitative study and our findings could not be generalizable, the findings should be extrapolated with caution. In addition, we explored challenges faced by HIV seronegative females in serodiscordant context; therefore, we had limitations in analysis of possible challenges faced by HIV seropositive females in the context of serodiscordant couples. Our results can only be used as comparative data or as the source of some hypothesis to be tested in clinical settings. 

## CONCLUSION

Overall, our study provided an in-depth perspective on numerous challenges faced by HIV-uninfected women in their lives in the context of HIV serodiscordant relationships. Also, this study supports the view of other investigators who believe that there is an urgent need for provision of appropriate counseling and empowerment programs for serodiscordant couples. The complexity of the challenges to which women are confronted in serodiscordant relationships needs to be understood by health care providers. Also, evidence on local conditions with global evidence can help the policymakers to design appropriate counseling and empowerment program for women in the context of HIV serodiscoedance and fight against AIDS. Developing policies that address their concerns can improve implementation of policies as well as provision of healthcare services for individuals. 

Considering the fact that the serodiscordant couples participating in this study are not a diverse and heterogeneous group and also their emotions, experiences and socio-cultural as well as structural challenges are different, further qualitative studies with larger sample size are required to address various gaps and unanswered issues left in this study as challenges associated with seeking support. It seems that there are differences between the challenges with which the positive and negative partners are faced in a serodiscordant relationship. It is recommended that a qualitative study should be conducted to understand the challenges of the HIV-infected married women whose husbands are not infected by HIV.
